# Effect of Methionine Oxidation and Substitution of α-Conotoxin TxID on α3β4 Nicotinic Acetylcholine Receptor

**DOI:** 10.3390/md16060215

**Published:** 2018-06-20

**Authors:** Jie Ren, Rui Li, Jiong Ning, Xiaopeng Zhu, Dongting Zhangsun, Yong Wu, Sulan Luo

**Affiliations:** 1 Key Laboratory of Tropical Biological Resources, Ministry of Education, Key Laboratory for Marine Drugs of Haikou, Hainan University, Haikou 570228, China; hndx2303@163.com (J.R.); flychcken@163.com (R.L.); ningjiong2018@163.com (J.N.); zhuxiaopeng@hainu.edu.cn (X.Z.); zhangsundt@163.com (D.Z.); wys211@163.com (Y.W.); 2 Institute of Tropical Agriculture and Forestry, Hainan University, Haikou 570228, China

**Keywords:** α-Conotoxin TxID, methionine oxidation and substitution, α3β4 nAChR, peptide synthesis and activity assay, molecular dynamics simulations

## Abstract

α-Conotoxin TxID was discovered from *Conus textile* by gene cloning, which has 4/6 inter-cysteine loop spacing and selectively inhibits α3β4 nicotinic acetylcholine receptor (nAChR) subtype. However, TxID is susceptible to modification due to it containing a methionine (Met) residue that easily forms methionine sulfoxide (MetO) in oxidative environment. In this study, we investigated how Met-11 and its derivatives affect the activity of TxID using a combination of electrophysiological recordings and molecular modelling. The results showed most TxID analogues had substantially decreased activities on α3β4 nAChR with more than 10-fold potency loss and 5 of them demonstrated no inhibition on α3β4 nAChR. However, one mutant, [M11I]TxID, displayed potent inhibition at α3β4 nAChR with an IC_50_ of 69 nM, which only exhibited 3.8-fold less compared with TxID. Molecular dynamics simulations were performed to expound the decrease in the affinity for α3β4 nAChR. The results indicate replacement of Met with a hydrophobic moderate-sized Ile in TxID is an alternative strategy to reduce the impact of Met oxidation, which may help to redesign conotoxins containing methionine residue.

## 1. Introduction

Conotoxins (Conopeptides, CTxs) are produced by marine cone snails belonging to the *Conus* genus, which are disulfide-constrained peptides targeting a range of ion channels and receptors [1,2,3,4]. α-CTxs are a subgroup of conotoxins characterized by their ability to inhibit nicotinic acetylcholine receptors (nAChRs) [5,6,7,8]. The nAChRs have been implicated in a range of diseases including pain, addiction, dementia, Parkinson’s disease, schizophrenia, obesity and cancer et al. [9,10]. As such, α-Ctxs are potential treatments for these difficult miscellaneous diseases.

**Figure 1:**
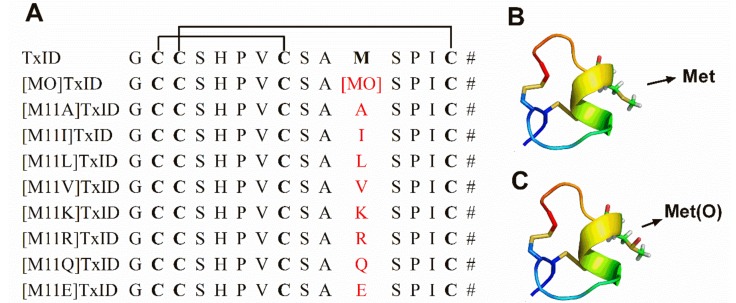
Sequences alignment of α-CTx TxID analogues with Cys (I–III, II–IV) disulfide bond connectivity (**A**) and structures of TxID (**B**) and [MO]TxID (**C**). (**A**) The sequences of TxID analogues are shown and the substituted residues at position 11 are highlighted in red. The # indicates an amidated C terminus; (**B**) Structural representation of TxID (PDB ID code 2M3I); (**C**) Spatial structure of [MO]TxID while Met residue is oxidized to MetO by addition of oxygen to its sulfur atom, which was produced by PyMOL.

A structurally novel α-conotoxin, TxID, was discovered by gene cloning in our lab from *Conus textile.* It consists of 15 amino acid residues possessing one readily oxidizable methionine at position 11 (Met-11) (Figure 1). TxID belongs to the α-4/6 conotoxin subfamily that potently blocks α3β4 nAChR subtype with high selectivity [11]. Differential sensitivity of α-CTx TxID on stoichiometry of α3β4 nAChR was examined previously. We used three α3:β4 RNA injection ratios of 1:1, 1:10 and 10:1 to form three different stoichiometry receptors of α3β4 subtype [12]. The results showed that inhibition of 1:10 injection ratio by TxID was comparable with regular 1:1 α3β4 nAChRs within 2-fold difference. However, potency of 10:1 injection ratio decreased 5-fold comparing with 1:1 α3β4 nAChR. TxID exhibited different sensitivity on different stoichiometry of α3β4 nAChRs, which could reflect different stoichiometries of α and β subunit [12]. To better understand which amino acids are responsible for the activity of TxID, we performed alanine scanning mutagenesis on all residues except for four Cys residues. TxID completely lost its inhibitory activity at α3β4 nAChR when five residues (i.e., His-5, Pro-6, Val-7, Met-11 and Pro-13) were mutated to alanine respectively. For [G1A]TxID, activity decreased nearly 19-fold relative to wild-type peptide. Other analogues, [S4A]TxID, [S9A]TxID, [S12A]TxID and [I14A]TxID almost maintained full potency at α3β4 nAChR. Our homology models and molecular dynamics(MD) simulations suggested that critical residues His-5, Pro-6 and Val-7 in loop 1 of TxID interacted with the α3 subunit, whereas Met-11 and Pro-13 located in loop 2 are close to β4 subunit [11,13]. These results reveal that Met is one of critical residues for binding α3β4 nAChR.

Methionine and cysteine are the only two sulfur-containing proteogenic amino acids and are extremely vulnerable to oxidation in vivo. Cystines are oxidized to form disulfide bonds that are crucial for structural and biological function of proteins. Oxidation of the amino acid methionine generates methionine sulfoxide (MetO) which may play a vital role in mediating the physiological function of some proteins [14]. With this information in mind, we investigated whether the oxidation of Met to MetO could alter the potency of α-CTx TxID at α3β4 nAChR subtype. Additionally, Met residue was substituted by other amino acids to ascertain the impact of postion 11 on TxID activity (Figure 1). In a series of electrophysiological experiments, we accessed activities of these analogues at various nAChR subtypes. Using electrophysiology assays combined with computer dynamic stimulations, we hoped to reveal the molecular mechanism of Met acting with α3β4 nAChR subtype.

## 2. Results

### 2.1. Peptide Synthesis

The native TxID and its derivatives were synthesized using Fmoc solid phase peptide synthesis with the side chains of Cys residues protected in pairs. Cys I and Cys III were protected by the acid-labile trityl (Trt) while Cys II and Cys IV were protected by the acid-resistant acetamidomethyl (Acm) [14]. Disulfide bonds with Cys (I–III, II–IV) connectivity of these peptides were formed using two-step oxidations. The folded peptide of TxID and its analogues were purified by HPLC. Electrospray-mass spectroscopy confirmed molecular mass of the folded peptides which was consistent with their corresponding theoretical values.

**Figure 2:**
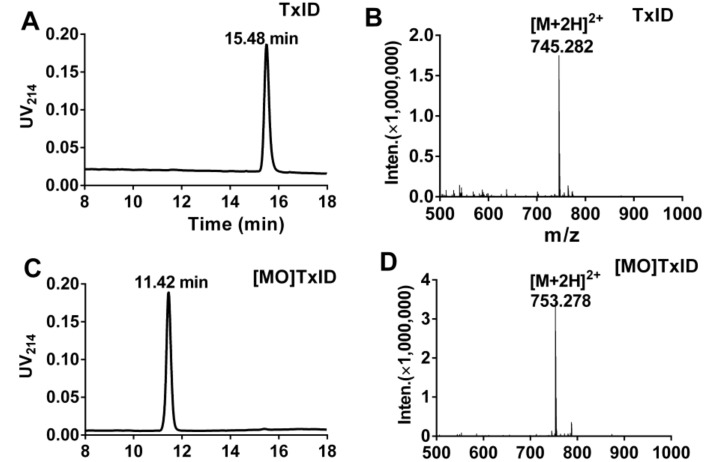
HPLC chromatograms and mass spectra of α-CTx TxID and [MO]TxID. Peptides were analyzed on a reversed phase analytical Vydac C18 (5 μm, 4.6 mm × 250 mm) HPLC column using a linear gradient of a 10–40% buffer B, and 90–60% buffer A over 20 min, where B = 0.05% TFA in 90% ACN; A = 0.075% TFA in water. The elution profile was monitored by measuring the absorbance at 214 nm. (**A**) HPLC chromatogram of α-CTx TxID; (**B**) Electrospray ionization mass spectrometry (ESI-MS) data for TxID with observed monoisotopic mass of 1488.56 Da; (**C**) HPLC chromatogram of [MO]TxID; (**D**) ESI-MS data for [MO]TxID with an observed monoisotopic mass of 1504.56 Da.

The [MO]TxID was synthesized through 10% hydrogen peroxide(H_2_O_2_) oxidation according to the reaction scheme of Methionine (TxID) + 10% H_2_O_2_ → Methionine Sulfoxide(TxID) + H_2_O. Then the oxidative product was purified by RP-HPLC. When methionine of TxID was oxidized to sulfoxide methionine, the hydrophilicity of [MO]TxID increased remarkably and its retention time shifted from 15.48 min to 11.42 min (Figure 2A,C). Meanwhile, the molecular weight of TxID and [MO]TxID was determined by ESI-MS respectively (Figure 2B,D). [MO]TxID showed a molecular weight increase of 16 Da, which was consistent with its theoretical mass (Figure 2B,D).

### 2.2. Characterization of [MO]TxID Effect on the α3β4 nAChR Subtype

**Figure 3:**
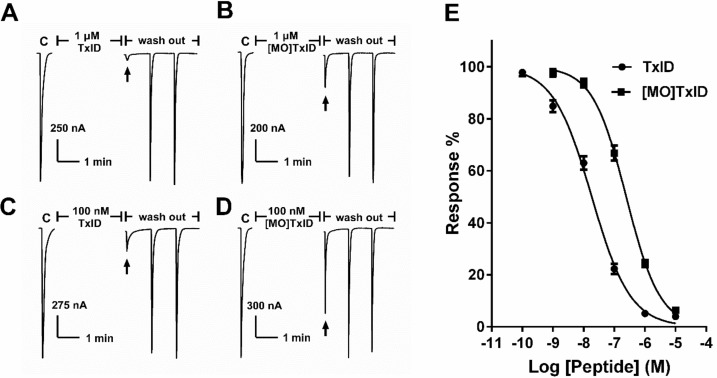
α3β4 nAChR-inhibitory activities of TxID and [MO]TxID. α3β4 nAChR was expressed in *Xenopus* oocytes as described under “Materials and Methods.” (**A**) TxID at 1 μM was added to the chamber and incubated with oocyte for 5 min. Then TxID was washed out and the response to 1-s pulses to ACh was again measured (arrow). “C” indicates control responses to Ach; (**B**) Representative ACh-evoked currents of rat α3β4 nAChR obtained in presence of [MO]TxID at 1 μM; (**C**) Representative ACh-evoked currents of rat α3β4 nAChR obtained that the oocyte was exposed to 100 nM TxID; (**D**) A representative response of one typical oocyte was exposed to 100 nM [MO]TxID; (**E**) Concentration-response analysis of the activities of TxID and [MO]TxID on rat α3β4 nAChR subtype. Error bars denote the means ± SEM. of the data from 3 to 8 separate oocytes.

The influence of Met oxidation of TxID on α3β4 nAChR subtype expressed in *Xenopus* oocytes was investigated. Representative traces of TxID and [MO]TxID on α3β4 nAChR are shown in Figure 3. At a concentration of 1 μM, TxID almost blocked 100% of ACh-evoked α3β4 nAChR-mediated currents whereas [MO]TxID only inhibited 72% of current amplitude (Figure 3A,B). At a concentration of 100 nM, TxID caused potent blockade of ACh-evoked currents (~80%), but [MO]TxID only inhibited 25% of current amplitude (Figure 3C,D). The concentration response curves indicated that TxID inhibited α3β4 nAChR with an IC_50_ of 18 nM (Figure 3E, Table 1). Remarkably, the IC_50_ value of [MO]TxID was 245 nM which was almost 13.3-fold less potent than that of TxID (Figure 3E, Table 1). These findings demonstrate that methionine is essential for TxID binding rat α3β4 nAChR. 

### 2.3. Effect of Mutants of Substitution at Met of TxID on α3β4 nAChR Subtype

**Figure 4:**
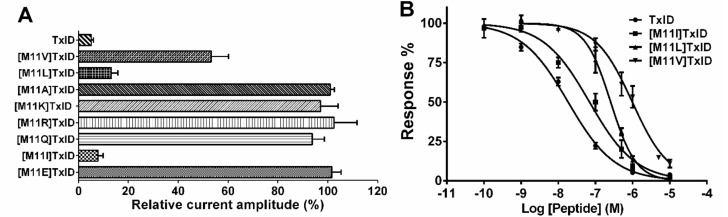
Inhibition of α3β4 nAChR by TxID and its Met substitution analogues. (**A**) Bar graph of normalized inhibition of ACh-evoked current is generated by methionine-substituted TxID analogues and native TxID. All peptides were tested at 1 μM and data are represented as mean ± SEM (*n* = 3–6); (**B**) Concentration-response curves are obtained for inhibition of α3β4 nAChR. Error bars denote the means ± SEM of the data from 3 to 8 separate oocytes.

The methionine of TxID was substituted by Ala, hydrophobic amino acids (Ile, Val and Leu) and charged amino acid (Lys, Arg, Glu and Gln) and these analogues (1 μM) were tested at α3β4 nAChR (Figure 4A). The [M11K]TxID, [M11R]TxID, [M11Q]TxID, [M11A]TxID and [M11E]TxID exhibited nearly no inhibition on α3β4 nAChR. Replacing the Met residue with Val resulted in a 40% decrease on blocking α3β4 nAChR. In contrast, [M11I]TxID and [M11L]TxID displayed comparable activity to the native peptide, which blocked >80% evoked current (Figure 4A).

The concentration-response relationships of all TxID analogues were determined (Figure 4B, Table 1). Three of them retained the potency on α3β4 subtype, and none of the analogues increased binding affinity compared to native peptide. The IC_50_ for [M11I]TxID on α3β4 nAChR subtype was 69.09 nM, which retained comparable activity compared to native peptide. Surprisingly, the activities of [M11L]TxID and [M11V]TxID reduced dramatically with an IC_50_ of 256.7 nM and 980.3 nM respectively (Table 1). There were 14-fold potency loss for [M11L]TxID and 53-fold potency loss for [M11V]TxID. We subsequently evaluated [M11L]TxID and [M11I]TxID on the other nAChR subtypes, including α6/α3β4, Mα1β1δε, α7, α9α10, α2β4, α3β2, α4β2 and α4β4. Only α6/α3β4 nAChR can be blocked with high concentration of 10 μM.

### 2.4. Homology Modeling and MD Simulations Demonstrate Potency Variation between TxID Analogues and α3β4 nAChR

**Figure 5:**
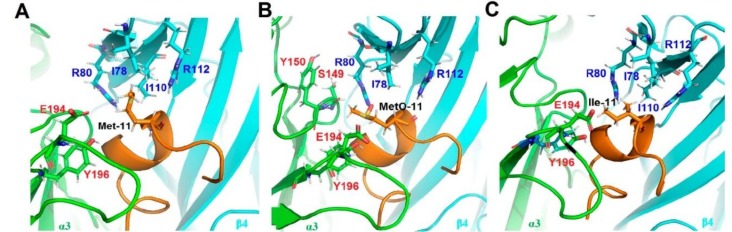
Molecular interactions between peptides and α3β4 nAChR through homology modeling and MD simulation. The α3 subunit is drawn in green, the β4 is in cyan, and the peptides are in brown. Amino acids around 4 Å radius of the Met and its substitutions are labeled. (**A**) The molecular model was shown between TxID and α3β4 nAChR during 50 ns MD simulations; (**B**) Snapshot at 50 ns of [MO]TxID and α3β4 interface; (**C**) Snapshot at 50 ns of [M11I]TxID and α3β4 interface.

Homology modeling/MD simulations were performed to understand the molecular mechanism for the activity of TxID analogues at α3β4 nAChR. Native TxID and its mutants bind to the extracellular domain of the rat α3β4 nAChR and the models demonstrate the binding pocket of peptides complexed with α3β4 nAChR (Figure 5). According to the model, Met-11 is surrounded by hydrophobic and charged amino acids in β4 subunit, including Ile78, Ile110, Arg112 and Arg80 (Figure 5A). Therefore, the substitution of Met by charged amino acids ([M11K]TxID, [M11R]TxID and [M11Q]TxID) would significantly impact the electrostatic interactions between peptides and receptors, which may result in a complete loss of inhibition at α3β4 nAChR . Through 50 ns dynamic stimulation, the distance between MetO and β4 subunit increases and MetO is impacted by more amino acids in α3 subunit, including Ser149, Tyr150 and Asp154 (Figure 5B). In addition, [MO]TxID is more hydrophilic than wildtype toxin, hence MetO would probably decrease hydrophobic interactions with Ile78 and Ile110. Besides, when Met residue was mutated to Ile residue, distance between two hydrophobic surfaces also increases, which partially weakens the interaction force of Ile-11 and receptors. Similarly, replacement of Met with Leu and Val leads to lose more potency on α3β4 nAChR.

## 3. Discussion

Cone snails have evolved many conopeptides which target to a wide variety of different voltage- or ligand-gated ion channels. The α-family conotoxins (α*-CTxs) act by blocking different isoforms of nAChRs, which locate in the neuromuscular junction or central nervous system broadly [5,6]. Methionine, sulfur-containing amino acid, was exist in different conotoxin families, such as α-, αD-, αO- and ω-CTxs (Table 2) [11,15,16,17,18,19,20,21,22,23,24,25,26,27,28]. Apart from TxID, several α-CTxs contain Met residue. SrIA and SrIB were discovered from the venom of *Conus spurius,* which can inhibit α4β2 and α1β1γδ nAChR, respectively [15]. EI demonstrates strong blockade of the nAChR subtypes of α4β2, α1β1γδ and α3β4 [15,16]. Additionally, methionine was detected in several αD-CTxs which formed dimer and bound different nAChR subtypes [25,26]. The ω-CTx MVIIA is a potent antagonist of N-type calcium ion channel (Cav2.2), which is the only cone snail-derived pharmaceutical drug. MVIIA contains a Met at the 12th position. Before it was developed as a drug candidate, hundreds of analogues of wild type peptide were tested. However, there was no ideal analogue substituting MVIIA, which indicated the difficulty to reform native peptide [29]. Pu14a containing Met at position 10 inhibits neuronal α3β2 and neuromuscular α1β1γδ nAChR [27]. Recently, a novel O-superfamily conotoxin, αO-GeXXVIIA, a disulfide-linked homodimer conopeptide, blocks α9α10 nAChR with high potency [28]. Therefore, the methionine possibly plays an important role in the modulation of the interaction between conotoxins and receptors. 

**Figure 6:**
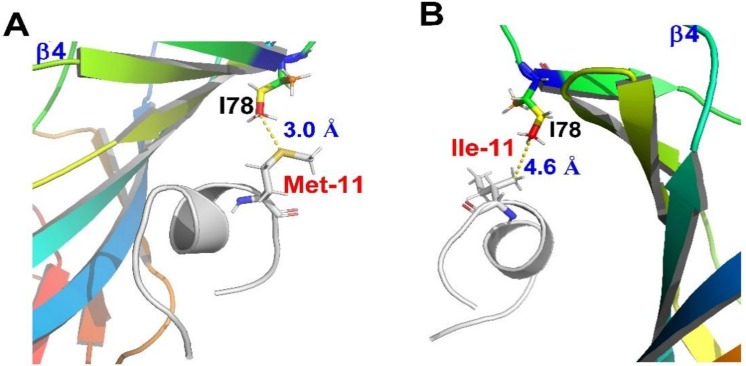
MD simulations demonstrate the distance difference between rat β4-I78 and Met-11 or Ile-11. (**A**) For native TxID, the distance between the sulfur atom of Met-11 and side-chain Cδ of β4-I78 lies within 3.5 Å; (**B**) For analogue [M11I]TxID, the distance between Cγ2 of Ile-11 and side-chain Cδ of β4-I78 is 4.6 Å.

Increasing evidences indicate that Met residue in proteins is the same as cysteine which serve as antioxidants and impact the structure of proteins [14]. Here, we investigated how methionine played a role in interaction between TxID and α3β4 nAChR. Firstly, [MO]TxID was synthesized and the potency on α3β4 nAChR was tested. It resulted in a 13.3-fold loss of activity compared to parent peptide. Previous study showed single amino acid substitutions in TxID shifted its selectivity. Analogously, Met oxidation in TxID probably change its specificity on α3β4 nAChR. To understand the role of Met in TxID, we systematically substituted Met residue by the other eight amino acids, including Ala, Ile, Val, Leu, Lys, Arg, Glu and Gln. Most analogues decreased or even lost the affinity at α3β4 nAChR. Only [M11I]TxID retained most activity on α3β4 nAChR (Table 1). Our findings show that [M11L]TxID binds with Hill coefficient of 1.27 and other TxID analogues bind with Hill coefficients less than 1 (0.69–0.88), which may predict the substitution of Met in TxID can allosterically regulate the conformation of α3β4 nAChR [30]. Consistent with the electrophysiology experiment results, the MD simulations data shows that Met residue can establish hydrophobic interactions with the residues in the binding pocket. In our models, the conversion of Met to MetO weakens the hydrophobic interaction between peptide and receptor. It is, therefore, not surprising to find the [MO]TxID have a decreased activity compared to TxID. Eight analogues were designed and synthesized. Their potencies were evaluated respectively to reveal effects of the 11th position amino acid of TxID on α3β4 nAChR. Among them, replacement of Met with Ile is only 3.8-fold less active against α3β4 nAChR. In the binding mode of [M11I]TxID at α3β4 nAChR, the spacing distance between position 11 and β4 (−)-I78 increased from 3.5 Å to 4.6 Å, which may slightly but not significantly weaken the hydrophobic interaction, which could be used to explain why [M11I]TxID has comparable activity relative to the parent peptide (Figure 6). 

To date, several α-CTxs and its analogues have been identified to antagonize α3β4 nAChR [11,31,32,33,34]. α4/6-CTx AuIB, from the venom of *Conus aulicus*, selectively inhibits α3β4 nAChR with an IC_50_ of 750 nM [31]. Alanine-scanning and MD simulations analysis revealed the side chain size, aromaticity, and hydrophobicity of phenylalanine (Phe) at position 9 can significantly affect the activity of AuIB at α3β4 nAChR [35]. α-CTx RegIIA was identified from *Conus regius* venom, inhibiting α3β2, α3β4, and α7 nAChRs [34]. Mutagenesis of RegIIA revealed α-CTx [N11A, N12A] RegIIA specific blocked α3β4 nAChR, then the molecular mechanism research suggested Asn-9, Asn-11, and Asn-12 involved in toxin-receptor interaction [36]. Another research elucidated specific β subunit residues interacting with RegIIA and AuIB [37]. Combining with our research results, the interaction between α-CTxs with α3β4 nAChR is complex, and further mutational and molecular mechanical studies are required to illuminate SAR between them.

Owing to its potent activity, the TxID has a potential in the development of a novel drug. Unfortunately, the conversion of Met to MetO is readily to happen during TxID synthesis and oxidation steps as well as under oxidative environment in vivo, which causes the loss of activity in the inhibition of α3β4 nAChR. Furthermore, conotoxins containing Met require low-temperature storage in the entire supply chain which increases the inconvenience and cost [38]. These drawbacks caused by Met should be considered in the pharmaceutical industry when transferring TxID to a drug. Hence, it is an ideal strategy that Met could be replaced by other stable amino acids or derivatives while maintaining its original activity. In this study, our research results showed substitution Met-11 with Ile retained comparable activity at α3β4 nAChR. Our research sheds a light on re-engineering conotoxins containing Met to retain their stability and activity.

In summary, we firstly investigated how the conversion of Met to MetO affected the efficacy of TxID at the α3β4 nAChR. The substitution of Met of TxID by an Ile maintained the comparable activity at the α3β4 nAChR. Homology models and dynamic simulation suggested the molecular mechanism of interaction between different analogues of TxID and α3β4 nAChR. These findings provide further information for understanding the function of Met residue in α-CTx TxID.

## 4. Materials and Methods

### 4.1. Chemical Synthesis and Oxidative Folding of α-Ctx TxID Analogues

The linear peptides of TxID and its mutants were synthesized as previously described [39]. Two disulfide bonds between cysteine residues form disulfide frameworks with regioselective protection method. Cysteine residues were protected with a pairwise combination of S-trityl on Cys I and Cys III and with S-acetamidomethyl on Cys II and IV. Acid-labile protecting groups Trt were cleaved from Cys I and Cys III during the acidic conditions, and the first disulfide bridge was formed by exposure to potassium ferricyanide (Ke3[Fe(CN)6]), then monocyclic peptides were purified by preparative RP-HPLC. The Acm protecting groups were subsequently removed and closed from Cys II and Cys IV by iodine oxidation. The synthesized peptides were purified on a Waters 2535 HPLC system (Milford, MA, USA) using preparative Vydac C18 column with a linear gradient of a 10–40% eluate B, and 90–60% eluate A over 30 min. Solvent B was 90% ACN, 0.092% TFA, and H_2_O; Solvent A was 0.1% TFA in H_2_O. The purity of the TxID and its analogues were determined by monitoring absorbance at 214 nm during HPLC (≥95% purity). ESI-MS mass spectrometry was utilized to confirm the identity of the products. 

### 4.2. Chemical Synthesis of [MO]TxID

Met is easily modified to methionine sulfoxide under oxidative conditions. α-CTx TxID (0.2 mg) firstly dissolved in 100 μL of 60% acetonitrile in ddH_2_O, which was incubated with 5 mL of 10% hydrogen peroxide (H_2_O_2_) in 20 min at room temperature. Then reaction products were separated using semi-preparative RP-HPLC with a linear gradient as above conditions. The purity of oxidative products was identified by Waters ACQUITY UPLC™ System with BEH300 C18 column (50 × 2.1 mm, 1.7 µm) (Waters Corporation, Milford, MA, USA). The molecular mass was measured on a Waters TQD mass spectrometer equipped with an electrospray ionization source (Waters Corporation, Milford, MA, USA).

### 4.3. RNA Preparation and Injection

Plasmid DNAs encoding rat nAChR subunits were kindly provided by Stefan H. Heinemann (Salk Institute, San Diego, CA, USA). The plasmids were linearized with appropriate restriction enzymes. Then capped RNA (cRNA) for the various subunits were made using transcription kit of the mMessage mMachine SP6 (Ambion, Austin, TX, USA) in vitro. The cRNA was purified using MEGAclear™ kit (Ambion). The concentration of each cRNA was determined using Smart Spec™ plus Spectrophotometer (Bio-Rad, Hercules, CA, USA) at 260 nm. cRNA of the various subunits was combined to give ~50 ng/μL of each subunit cRNA. 50.6 nL of this mixture was injected into each *Xenopus* oocyte with a Drummond microdispenser (Drummond Scientific, Broomall, PA, USA), and incubated at 17 °C. Oocytes were injected within 1 day of harvesting, and recordings were made 2–4 days post-injection.

### 4.4. Voltage-Clamp Recording

Oocytes were voltage-clamped and exposed to ACh and CTxs as described previously [40]. Briefly, oocytes were transferred into the recording chamber (~50 μL in volume) and gravity-perfused at 2 mL/min with ND96 buffer (96 mM NaCl, 2.0 mM KCl, 1.0 mM MgCl_2_·6H_2_O, 1.8 mM CaCl_2_·2H_2_O, 5 mM HEPES, pH 7.1–7.5) containing 1 μM atropine and 0.1 mg/mL bovine serum albumin (BSA). For α9α10 subtype, the ND96 contained no atropine. Two electrode voltage clamp recordings from oocytes were carried out at room temperature using an Axoclamp 900A amplifier (Molecular Devices Corp., Sunnyvale, CA, USA) at a holding potential of −70 mV. The continuous gravity perfused with standard ND96 solution and stimulated with 1-s pulses of ACh once every minute. For screening of receptor for toxin concentration 10 μM and lower, once a stable baseline was achieved, we added 5 μL of different concentration toxin to the chamber and waited for 5 min, then applied perfusion system, during which 1-s pulses of 100 μM ACh were applied every minute until a constant level of block was achieved. The electrophysiology data were recorded and analyzed using Clampfit 10.2 software (Molecular Devices Corp., Sunnyvale, CA, USA). The results were acquired with at least three oocytes. 

### 4.5. Statistical Analysis of Data

The data and statistical analysis in this study comply with previous strategy [12]. An average of five control responses just preceding a test response was used to normalize the test response to obtain “% response”. All electrophysiological data were collected at least three oocytes and represent means ± standard error of the mean (SEM). The dose-response data were fit to the equation, % response = 100/{1 + ([toxin]/IC_50_)^nH^}, where n_H_ is the Hill coefficient, by nonlinear regression analysis using GraphPad Prism 5.0 (GraphPad Software, San Diego, CA, USA).

### 4.6. Homology Modeling

Homology models of the extracellular ligand binding domain of Rat α3β4 nAChR were generated using Modeler9v14 as previous method [13,41]. The co-crystal structure of Ac-AChBP (*Aplysia californica* acetylcholine-binding protein) with the potent mutant TxIA(A10L) (PDB ID: 2BR8) [42] and crystallographic structure of human α9 subunit (PDB ID: 4D01) [43] were adopted as structural template. These templates were chosen to generate model of the interaction between toxin and rat α3β4 nAChR ligand binding domains. Then the model was selected for subsequent molecular dynamics (MD) simulations and analysis.

### 4.7. Molecular Dynamics Simulations

Molecular dynamics (MD) simulations were performed for the α3β4 nAChRs with TxID and its two analogues. The structure of methionine sulfoxide was built with GaussView V.5 and Amber tools 14. These models were implemented using the GROMACS5.1 [44] with the amber ff99SB-ILDN30 force field [45]. The MD parameters complied with previous study. 50 nanoseconds simulation was performed for each model and graphics were produced using PyMOL package (http://www.pymol.org/).

## Figures and Tables

**Table 1:** IC_50_ and Hill slope values for blockade of α3β4 nAChR subtype by TxID analogues.

TxID Analogues	α3β4 nAChR, IC_50_ *	Hill Slope *	IC_50_ Ratio Relative to TxID
TxID	18.38 (15.35–22.0)	0.69 (0.61–0.77)	1
[MO]TxID	245.0 (211.7–283.6)	0.79 (0.71–0.87)	13.3
[M11V]TxID	980.3 (760.1–1264)	0.88 (0.68–1.09)	53.3
[M11I]TxID	69.09 (50.68–94.19)	0.70 (0.57–0.83)	3.8
[M11L]TxID	256.7 (211.7–311.2)	1.27 (1.01–1.54)	14.0
[M11K]TxID	>10,000 *^a^*	-	-
[M11R]TxID	>10,000 *^a^*	-	-
[M11Q]TxID	>10,000 *^a^*	-	-
[M11E]TxID	>10,000 *^a^*	-	-
[M11A]TxID	>10,000 *^a^*	-	-

* Numbers in parentheses are 95% confidence intervals. *^a^* Less than 50% block at 10 μM.

**Table 2:** Sequences and receptor activities of conotoxins comprising Met residue. #, amidated C–terminus; γ, γ–carboxyglutamate; O, 4–trans–hydroxyproline; Z, pyroglutamate. The Met residues are in bold.

Peptide	Species	Sequence	Activities	Reference
α-TxID	*C. textile*	GCCSHPVCSAMSPIC#	α3β4 > α6/α3β4 > α2β4	[11]
α-SrIA	*C. spurius*	RTCCSROTCRMγYPγLCG#	α1β1γδ, α4β2	[15]
α-SrIB	*C. spurius*	RTCCSROTCRMEYPγLCG#	α1β1γδ, α4β2	[15]
α-EI	*C. ermineus*	RDOCCYHPTCNMSNPQIC#	α1β1γδ, α3β4, α4β2	[15,16]
ω-CnVIIA	*C. consors*	CKGKGAOCTRLMYDCCHGSCSSSKGRC#	Ca_2.2_ > Ca_2.1_	[17]
ω-CVIB	*C. catus*	KGKGASCRKTMYDCCRGSCRSGRC#	Ca_2.2_~Ca_2.1_ > Ca_2.3_	[18]
ω-CVIC	*C. catus*	CKGKGQSCSKLMYDCCTGSCSRRGKC#	Ca_2.1_~Ca_2.2_	[18]
ω-CVID	*C. catus*	CKSKGAKCSKLMYDCCSGSCSGTVGRC#	Ca_2.2_ > Ca_2.1_	[18]
ω-MVIIA	*C. magus*	CKGKGAKCSRLMYDCCTGSCRSGKC#	Ca_2.2_ > Ca_2.1_	[19,20,21]
ω-MVIIC	*C. magus*	CKGKGAPCSKTMYDCCSGSCGRRGKC#	Ca_2.1_ > Ca_2.2_	[21,22]
ω-MVIID	*C. magus*	CQGRGASCRKTMYNCCSGSCNRGRCC#	Ca_2.1_ >> Ca_2.2_	[23,24]
αD-VxXXA	*C. vexillum*	DVQDCQVSTOGSKWGRCCLNRVCGPMCCPASHCYCVYHRGRGHGCSC (dimer)	N.D.	[25]
αD-VxXXB	*C. vexillum*	DDγSγCIINTRDSPWGRCCRTRMCGSMCCPRNGCTCVYHWRRGHGCSCPG (dimer)	α7, α3β2, α4β2	[25]
αD-VxXXC	*C. vexillum*	DLRQCTRNAPGSTWGRCCLNPMCGNFCCPRSGCTCAYNWRRGIYCSC (dimer)	N.D.	[25]
αD-cap	*C. capitaneus*	EVQECQVDTPGSSWGKCCMTRMCGTMCCSRSVCTCVYHWRRGHGCSCPG (dimer)	α7, α3β2, α4β2	[26]
αD-mus	*C. mustelinus*	DVRECQVNTPGSKWGKCCMTRMCGTMCCARSGCTCVYHWRRGHGCSCPG	α7, α3β2, α4β2	[26]
Pu14a	*C. pulicarius*	DCPPHPVPGMHKCVCLKTC	α3β2, α6α3β2	[27]
O-GeXXVIIA	*C. generalis*	ALMSTGTNYRLLKTCRGSGRYCRSPYDCRRRYCRRISDACV	α9α10, α1β1εδ	[28]
